# MiR-891a-5p as a prognostic marker and therapeutic target for hormone receptor-positive breast cancer

**DOI:** 10.7150/jca.40750

**Published:** 2020-04-06

**Authors:** Zhiqiang Zhang, Lu Xu, Lijie He, Jin Wang, Xiaonan Shi, Zhi Li, Sha Shi, Kezuo Hou, Yuee Teng, Xiujuan Qu

**Affiliations:** 1 Department of Medical Oncology and Key Laboratory of Anticancer Drugs and Biotherapy of Liaoning Province, The First Hospital of China Medical University, Shenyang 110001, China; 2 Department of Medical Oncology, Liaoning Provincial People's Hospital, The People's Hospital of China Medical University, Shenyang 110016, China; 3 Department of Medical Oncology, the First Hospital of Zhengzhou University, Zhengzhou 450052, China

**Keywords:** breast cancer, microRNAs, prognosis, miR-891a-5p, miR-383-5p, metastasis

## Abstract

**Background**: Breast cancer is one of the most frequent malignant tumors worldwide, with 1.67 million newly-diagnosed cases and 522,000 deaths each year. Therefore, seeking the novel biomarkers and therapeutic targets that contribute to postoperative recurrence and metastasis in patients with breast cancer is emerging and facilitates the development of innovative therapeutics.

**Methods**: Retrieving the dataset of patients with hormone receptor (HR)-positive breast cancers from Gene Expression Omnibus (GEO) and collecting the data from the patients with HR-positive breast cancers enrolled in the First Affiliated Hospital of China Medical University are so as to identify the miRNAs associated with metastasis and distant metastasis-free survival (DMFS). Then MTT and Transwell migration assays were used to validate the effect of miRNAs on cell proliferation and migration of estrogen receptor-positive breast cancer T47D and MCF7 cells *in vitro*, respectively.

**Results**: From GSE59829 dataset, the miRNA expression levels of miR-891a-5p, miR-383-5p and miR-1295a were significantly downregulated while the levels of miR-128-3p, miR-661 and miR-296-3p were significantly upregulated in breast cancers from patients with metastasis as compared to the matched non-metastatic group. Moreover, low expression levels of miR-891a-5p, miR-383-5p and miR-1295a or high expression levels of miR-128-3p, miR-661 and miR-296-3p were respectively associated with low DMFS in patients with breast cancer. Our clinical cohort study supported that the levels of miR-891a-5p, miR-383-5p and miR-1295a were significantly lower in breast cancers from the metastasis group when compared with non-metastatic group. However, there is no significant difference with regard to the levels of miR-128-3p, miR-661 and miR-296-3p in breast cancer between these two groups. Moreover, low expression levels of miR-891a-5p and miR-383-5p but not miR-1295a in breast cancer were significantly associated with low DMFS in patients, implying that the expression of miR-891a-5p and miR-383-5p were the potential prognosis markers for metastatic human breast cancers. Further investigation disclosed that miR-891a-5p but not miR-383-5p restrained both proliferation and migration of T47D and MCF7 cells. In silico analysis of miRNAs target gene through online computational algorithms revealed that A Disintegrin and metalloproteinase domain-containing protein 10 (ADAM10) is the downstream target for miR-891a-5p. Further study confirmed that miR-891a-5p impeded ADAM10 expression by directly binding to its 3'UTR, leading to the inhibition of breast cancer cells proliferation and migration. Moreover, silencing ADAM10 inhibited T47D and MCF7 cells growth and migration.

**Conclusion**: miR-891a-5p is the vital prognostic marker for HR-positive breast cancer. In addition, miR-891a-5p and miR-383-5p are the potential targets for HR-positive breast cancer therapeutics.

## Introduction

Breast cancer is one of the most frequent malignant tumors worldwide, with 1.67 million newly-diagnosed cases and 522,000 deaths each year 1. Most breast cancers (70% of cases) were hormone receptor (HR)-positive 2. Although the patients with HR-positive and early-stage breast cancer had a longer survival time after receiving endocrine treatment, up to 50% of these patients did not respond because of primary or acquired resistance to the treatment 3, 4. Given the heterogeneity of breast cancer, it is vital to find out the biomarker for prognosis so as for early intervene.

It is reported that multigene prognostic tests are beneficial for predicting prognosis of HR-positive breast cancers due to their accuracy. The first-generation approaches for multigene prognostic tests including Oncotype DX (21 genes) 5, MammaPrint (70 genes) 6, Genomic Grade Index (97 genes) 7 are accurate to predict early recurrence 8. Recently developed tests such as Prosigna (PAM50, 58 genes) 9, EndoPredict^10^, Breast Cancer Index[11]are proved as better prognostic tools for both early and late recurrences. However, these tests are either expensive or insensitive to predict distant metastasis. Therefore, seeking the novel biomarkers and therapeutic targets that contribute to postoperative recurrence and metastasis in patients with HR-positive breast cancer is emerging and facilitates to develop innovative therapeutics.

MicroRNAs (miRNAs) are wide-distributed, single-stranded and non-coding RNA molecules. They were involved in the regulation of gene expression by binding to the 3'-untranslational region (UTR) of target genes and dampening the translation of mRNAs 12. Compared to mRNA, miRNA is relatively stable and tolerance to the ribonuclease. miRNAs in formalin-fixed paraffin-embedded specimens (FFPEs) and body fluid can be fully preserved, which have been discovered to detect and predict prognosis in patients with cancer^13^, ^14^.

Therefore, in this study, upon retrieving the dataset of patients with HR-positive breast cancers from Gene Expression Omnibus (GEO) and collecting the data from the patients with HR-positive breast cancers enrolled in the First Affiliated Hospital of China Medical University, we would like to identify the miRNAs associated with metastasis and distant metastasis-free survival, and then characterize the underlying mechanism for this miRNA in regulation of breast cancer cells proliferation and migration.

## Materials and Methods

### Retrieval of the miRNAs expression profile

On the GEO website, the terms “breast cancer” AND “miRNA” AND “metastasis” were input to search the appropriate data series for the current study. Meanwhile, miRNA expression profiles were tracked and analyzed using the original information from GEO database. The criteria for selection of dataset are as follow.(1) Human breast cancer; (2) ER-positive and HER-2 negative; (3) Metastasis; (4) Clinical grouping studies in GEO, focusing on GSE series (including design-related clinical studies of GSM dataset); (5) The number of samples was at least higher than 30 cases; (6) Triple-negative breast cancer and HER-2 positive breast cancer were excluded. Consequently, the dataset GSE59829 was located.

The original data were retrieved from the GEO database of NCBI (www.ncbi.nlm.nih.gov/geo), and the access number is GSE59829. A total of 1146 miRNAs were detected on the platform (>97%, includes all of the miRNAs in microBase 12.0), and the access number was GPL8179.

### Analysis of miRNAs expression profile

Subgroup analysis was further carried out according to the purpose of this study. Firstly, 92 cases of patients with ESR1 (+) and ERBB2 (-) early breast cancer (T1-T2N0M0) were screened out from 123 breast cancer patients. In order to eliminate the influence of T2 stage on distant metastasis and find out the correlation between the expression of miRNAs and distant metastasis in patients with smaller primary breast cancer (T1N0M0), 38 patients with T2N0M0 were excluded from this study. Consequently, the data from 54 cases of patients with T1N0M0, ESR1(+)/ERBB2(-) breast cancer were used in this study. Among them, 21 patients had distant metastasis. The median follow-up time was 26 months (9-57 months). There were 33 cases of patients in the control group without recurrence or metastasis during the follow-up period. The median follow-up period was 116 months (71-172 months). After transforming the common names of probes and miRNAs, R software (version.3.6.0) was used for statistical analysis to compare the expression level of miRNAs between these two groups.

### Patients

The follow-up time of the study group (distant metastasis group) was the time from postoperative to distant metastasis. From January 2000 to April 2015, 29 patients with HR-positive early-stage (T1-2N0M0) breast cancer with distant metastasis after surgery in the First Affiliated Hospital of China Medical University were selected according to the inclusion criteria. One patient was excluded from the group because of the low concentration of total RNA after FFPE extraction. Twenty-eight patients were finally selected. The follow-up time was 6-86 months, and the median follow-up time was 46 months. Among them, 19 patients received adjuvant chemotherapy and 27 patients received adjuvant endocrine therapy. The patients (n=34) in the control group were those who had no recurrence and metastasis after surgery. The follow-up time was ranged from 59 to 131 months, and the median follow-up time was 81 months. Among them, 20 patients received adjuvant chemotherapy and 34 patients received adjuvant endocrine therapy.

### Quantitative real-time polymerase chain reaction (qRT-PCR)

Total RNA of formalin-fixed paraffin-embedded (FFPE) specimens from 34 patients without metastasis and 28 patients with distant metastasis or breast cancer cells was extracted using miRNeasy FFPE kit (GIAGEN, USA) per manufacturer's instructions. Then mRNA was transcribed into cDNA using Mir-x miRNA First-Strand Synthesis Kit (639522, Takara Biotechnology Co., Ltd., Dalian, China). Fluorescence quantitative PCR was utilized to detect the expression level of the targeted genes using cDNA as templates. The relevant expression level of *miRNAs* was normalized to *U6 small nuclear RNA*. The primers (5'-3') were: hsa-miR-128-3p: TCACAGTGAACCGGTCTCTTGAA; hsa-miR-661: TGCCTGGGTCTCTGGCCTAAAA; hsa-miR-296-3p: GAGGGTTGGGTGGAGGCTAAAA; hsa-miR-891a-5p: TGCAACGAACCTGAGCCACTA; hsa-miR-383-5p: AGATTAGAAGGTGATTGTGGGGG; hsa-miR-1295a: ATAGGCCGCAGATCTGGGTAA. The primers for U6 small nuclear RNA were: forward: GCTTCGGCAGCACATATACTAAAAT; reverse: CGCTTCACGAATTTGCGTGTCAT. Reactions included 6μl RNase Free dH_2_O, 2μl cDNA/DNA, 0.8μl primers, 0.8 μl PCR reverse primer, 0.4 μlROX Reference Dye IIand 10μlSYBR Premix Ex Taq II and were conducted using the following temperature cycles: 95°C 30 sec, 95°C 5 sec for 40 cycles, and 58°C (extension) 34 sec.

### Materials

Primary antibody against ADAM10 was obtained from ProteinTech Company. Primary antibody against β-actin and secondary antibodies against HRP-linked anti-rabbit IgG were purchased from Solarbio Company. Small interfering RNA (siRNA) and miRNA were synthesized by Genepharma company (Shanghai, China). The sequences were as follows: siADAM10 (5'-CCAGCAGAGAGAUAUAUUATT-3'); Negative control siRNA (5'-UUCUCCGAACGUGUCACGUTT-3'); miR-NC (5'-CAGUACUUUUGUGUAGUACAA-3'), hsa-miR-891a-5p mimic (5'-UGCAACGAACCUGAGCCACUGAAGUGGCUCAGGUUCGUUGCAUU-3'), hsa-miR-891a-5p inhibitor (5'-UCAGUGGCUCAGGUUCGUUGCA-3'),hsa-miR- 383-5p mimic (5'-AGAUCAGAAGGUGAUUGUGGCUCCACAAUCACCUUCUGAUCUUU-3'), hsa-miR-383-5p inhibitor (5'-AGCCACAAUCACCUUCUGAUCU-3').

### Cell culture

T47D and MCF7 breast cancer cells were obtained from American Type Culture Collection (ATCC) and cultured in Dulbecco's modified Eagle's medium (DMEM, Gibco, Grand Island, USA) containing 10% fetal bovine serum (FBS, Gibco, Grand Island, USA), 100 U/ml penicillin and 100 μg/ml streptomycin (Millipore, Darmstadt, Germany). The cells were maintained in a humidified incubator at 37℃ with 5% CO_2_.

### Transfection

Breast cancer cells (2x10^5^) were seeded in 6-well plate for 24 hrs. Then miR-NC, miR-891a-5p mimic, miR-891a-5p inhibitor, miR-383-5p mimic, miR-383-5p inhibitor, siADAM10, or negative control siRNA was transfected into the cells by using Lipofectamine 2000 reagent (GIBCOBRL, MD, USA) for indicated times according to the manufacturer's instructions. Then the transfected cells were used for qPCR, western blot, luciferase activity, cell viability, and migration assays.

### Cell viability determination

Cell viability was determined using MTT assay. Briefly, the cells (3,000) were seeded in 96-well plate. After treatment of cells with various conditions, the medium was removed and 25 ul of MTT (5 mg/ml, Sigma) was added to each well for 4 hrs incubation at 37 degrees. Then 200 ul of DMSO was added to each well, and the absorbance at 490 nm was read using microplate reader.

### Transwell migration assay

Cells migration was assessed using BD Falcon™ Cell Culture Inserts (353,097, CA, USA) according to the manufacturer's instructions. Briefly, after treatment of cells with various conditions, the cells were respectively trypsinized and resuspended in serum-free medium. Cells (1 × 10^5^) with the serum-free medium were plated in the insert and allowed to migrate through a semi supermeable (8 μm pore size) membrane toward 30% FBS medium in the bottom chamber for 24 hrs. After that, unmigrated cells were removed with swab from the top of the membrane and subsequently fixed with methanol and stained with crystal violet. The images of migrated cells at the bottom of the membrane were captured using an inverted microscope CK40 (Olympus, Japan). 5 areas of the membrane were captured for each sample.

### Western blot analysis

After cells had been treated with different conditions, cell lysates were extracted by using lysis buffer (BC3711, Solarbio) containing 1 mM phenylmethylsulfonyl fluoride (PMSF) and protease cocktail inhibitor. Protein concentration was determined by BCA protein assay reagent (Solarbio). Forty µg of total protein was resolved by an8% SDS-PAGE and transferred to PVDF membrane (Thermo Fisher Scientific). After blocking with 5% non-fat milk (Solarbio), the membrane was incubated with primary antibody mentioned in Materials overnight at 4 ^o^C. Subsequently, the membrane was incubated with secondary antibody for 90 minutes at room temperature. Western ECL substrate (PIERCE) was used to develop the immunoblot.The results were quantified by plotting the density of the band using Gel-Pro-Analyzer Software.

### Dual-Luciferase Reporter Assay

To generate the pmirGLO-ADAM10-3'UTR reporter plasmid, the human ADAM10-3'UTR region (423 bp) encompassing the binding site of miR-891a-5pwas amplified using PCR primers (3'UTR-F: 5'-GCAGCTAGCACTAAACCCTCACAAG-3'; 3'UTR -R: 5'-TTGCGTCGACACAGAAGTACAGTGTA-3') and then cloned into the luciferase vector pmirGLO(Promega) using NheI and SalI restriction enzymes (New England Biolabs). T47D cells were transfected with different combinations including pmirGLO-ADAM10-3'UTR (WT) plus miR-NC; pmirGLO-ADAM10-3'UTR (WT) plus miR-891a-5p; pmirGLO-ADAM10-3'UTR (Mut) plus miR-NC; pmirGLO-ADAM10-3'UTR (Mut) plus miR-891a-5p for 48 hrs. Then the cells were harvested and lysed, and luciferase activity was detected by GloMax®- Multi Detection System (Promega) using the Dual- Luciferase Reporter Assay Kit (E1910, Promega). Luciferase activity was normalized to renilla luciferase activity.

### Immunohistochemistry

Formalin-fixed, Paraffin-embedded (FFPE) tumor tissue blocks were sectioned into 3-µm slices, and immunohistochemistry staining was performed using SP Immunohistochemistry Kit (Solarbio) per manufacturer's instruction. Briefly, the sections were deparaffnized, dehydrated, antigen retrieval, blocking and incubated with anti-ADAM10 (ProteinTech) and secondary antibodies sequentially. Immune complex visualization was developed using 3, 30-diamino-benzidine tetrahydrochloride (DAB kit, ProteinTech). Semiquantitative scoring criterion was used to evaluate the expression of ADAM10 in the membrane and cytoplasm of the cells. The staining intensity of cancer cells was graded on a scale of 0-3: 0 (no staining), 1 (light yellow), 2 (brown staining), 3 (heavy brown staining). The proportion of stained cells was recorded as (0-3): 0-5% (0), 6-25% (1), 26-50% (2), 51-75% (3), and 76-100% (4). Taken together, the ADAM10 protein expression was assessed as follows: negative (-), score = 0; weak expression (+), score =1-4; moderate expression (++), score = 5-8; and strong expression (+++), score = 9-12.

### Statistics

Microarray analysis of miRNAs expression profiles is based on bioinformatics statistical methods, and statistical analysis is carried out by R software running R language. Bioconductor.org/biocLite.R (limma, input), pheatmap, and other packages were installed. After normalizing the original matrix data of GSE59829, miRNAs data were analyzed. Thermal maps and other expression profiles were produced. After screening by bioinformatics methods, the data were analyzed by IBM SPSS Statistics Version 19.0. The endpoint of survival analysis was distant metastasis-free survival (DMFS). The survival curve was drawn by Kaplan-Meier method, and survival difference was analyzed by log-rank test. T-test was used for comparison between test groups and P < 0.05 was considered as statistical significance.

## Results

### Identification of miRNAs involved in the metastasis of human estrogen receptor-positive breast cancers

In order to identify the miRNAs which contribute to the metastases of human breast cancers, microarray data representing the ESR1(+)/ERBB2(-) breast cancer specimens from 54 patients was retrieved from Gene Expression Omnibus (GEO, accession GSE59829)^15^ so as to compare the expression levels of miRNAs of specimens between the patients with or without metastasis. As shown in **Figure S1**, top 20 miRNAs were firstly characterized based on their differential expression levels between non-metastasis and metastasis groups using R software. Top 6 miRNAs were further identified according to the fold change levels level between non-metastasis and metastasis groups by SPSS software (**Figure S1**). In specific, the miRNA expression levels of miR-891a-5p, miR-383-5p and miR-1295a were significantly downregulated in cancer tissues from patients with metastasis as compared to the matched non-metastatic group (**Figures S2A, S2B, and S2C**). Conversely, the levels of miR-128-3p, miR-661 and miR-296-3p were significantly upregulated in cancers from patients with metastasis when compared with matched non-metastatic group (**Figures S2D, S2E, and S2F**). Moreover, low expression levels of miR-891a-5p, miR-383-5p and miR-1295a or high expression levels of miR-128-3p, miR-661 and miR-296-3p in breast cancer tissues were associated with low distant metastasis-free survival (DMFS) in patients with breast cancer (**Figure S3**).

### miR-891a-5p and miR-383-5p as prognosis markers for metastatic human breast cancers

**Figure 1:**
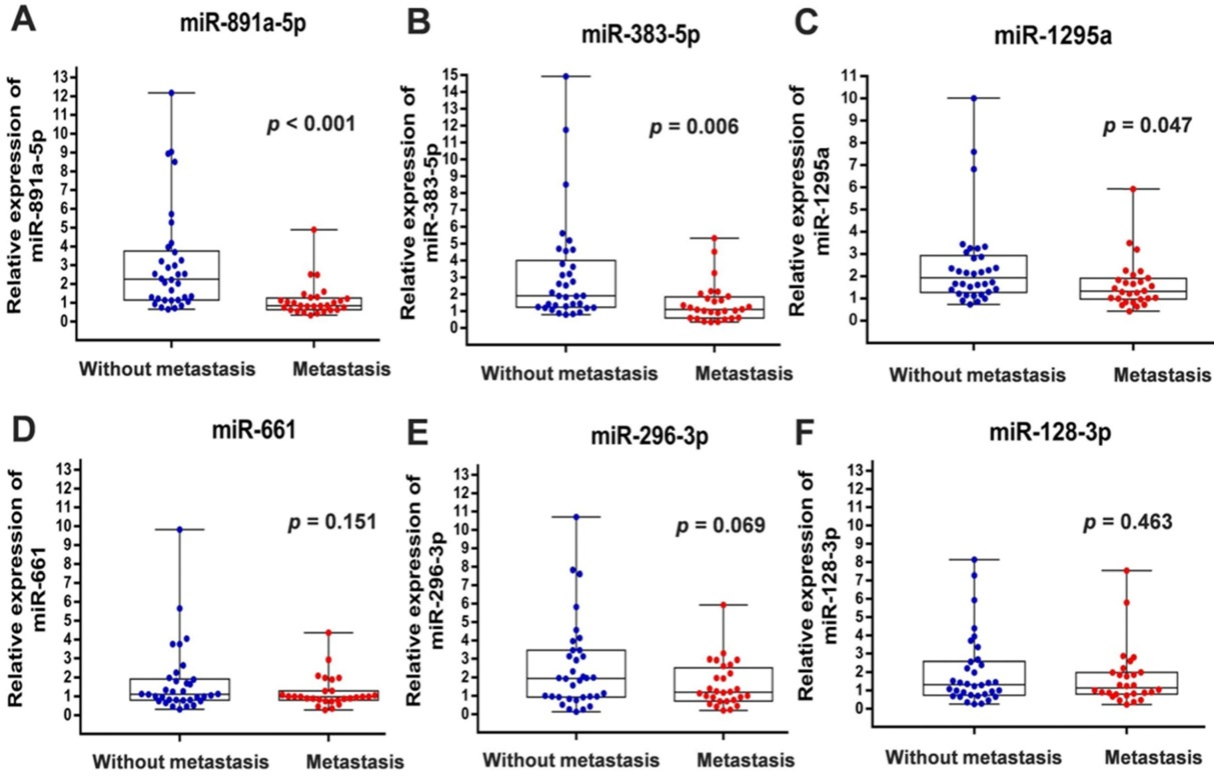
** Differential expression of miRNAs in breast cancers from patients with or without metastasis enrolled in the First Affiliated Hospital of China Medical University.** (A) miR-891a-5p; (B) miR-383-5p; (C) miR-1295a; (D) miR-661; (E) miR-296-3p; (F) miR-128-3p. Non-metastasis group: n=34; Metastasis group: n=28.

To verify the above findings, we collected the cancer specimens and follow-up data from patients with breast cancer who were enrolled in the First Affiliated Hospital of China Medical University. Total RNA of specimens from 34 patients without metastasis and 28 patients with distant metastasis were extracted for real-time PCR analysis. In line with the results from GSE59829 dataset, our investigation showed that the miRNA levels of miR-891a-5p, miR-383-5p and miR-1295a were significantly decreased in cancer tissues from patients with metastasis compared to the matched non-metastatic group (**Figures 1A, 1B and 1C**). However, there is no significant difference with regard to the levels of miR-128-3p, miR-661 and miR-296-3p in breast cancer tissues between these two groups (**Figures 1D, 1E and 1F**).

**Figure 2:**
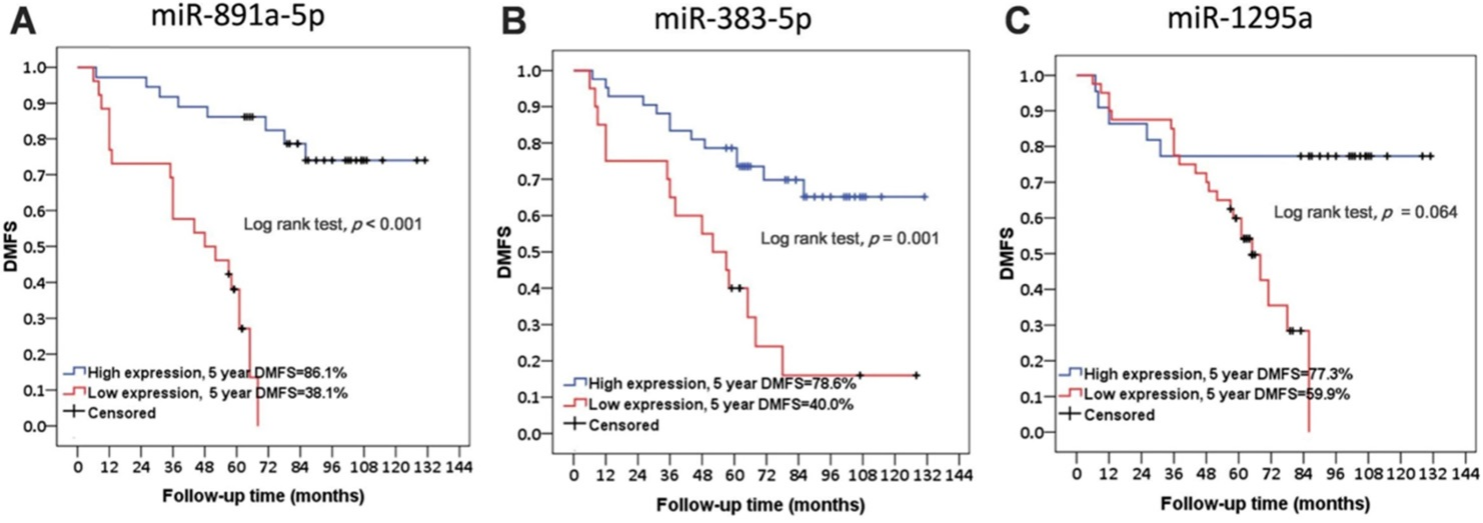
** The correlation between the expression level of miRNAs in cancer tissues and distant metastasis-free survival in patients with breast cancer from the First Affiliated Hospital of China Medical University.** (A) miR-891a-5p. (B) miR-383-5p. (C) miR-1295a.Non-metastasis group: n=34; Metastasis group: n=28.

Further, basic characteristics including the age and disease stage of patient, the receiving adjuvant therapies, and the molecular subtypes and histotypes of cancers were not significantly different in between patients with or without metastasis (**Table 1**). Interestingly, the difference regarding the time for follow-up of the patient between these two groups was at a significant level (**Table 1**). As presented in **Figure 2**, low expression levels of miR-891a-5p and miR-383-5p but not miR-1295a in breast cancer tissues were significantly associated with low distant metastasis-free survival (DMFS) in patient with breast cancer.

These results signified that the expression of miR-891a-5p and miR-383-5p was the potential prognosis marker for metastatic human breast cancers, although a larger tumor sample size is required for a definitive conclusion.

### miR-891a-5p and miR-383-5p inhibited breast cancer cells proliferation

**Figure 3:**
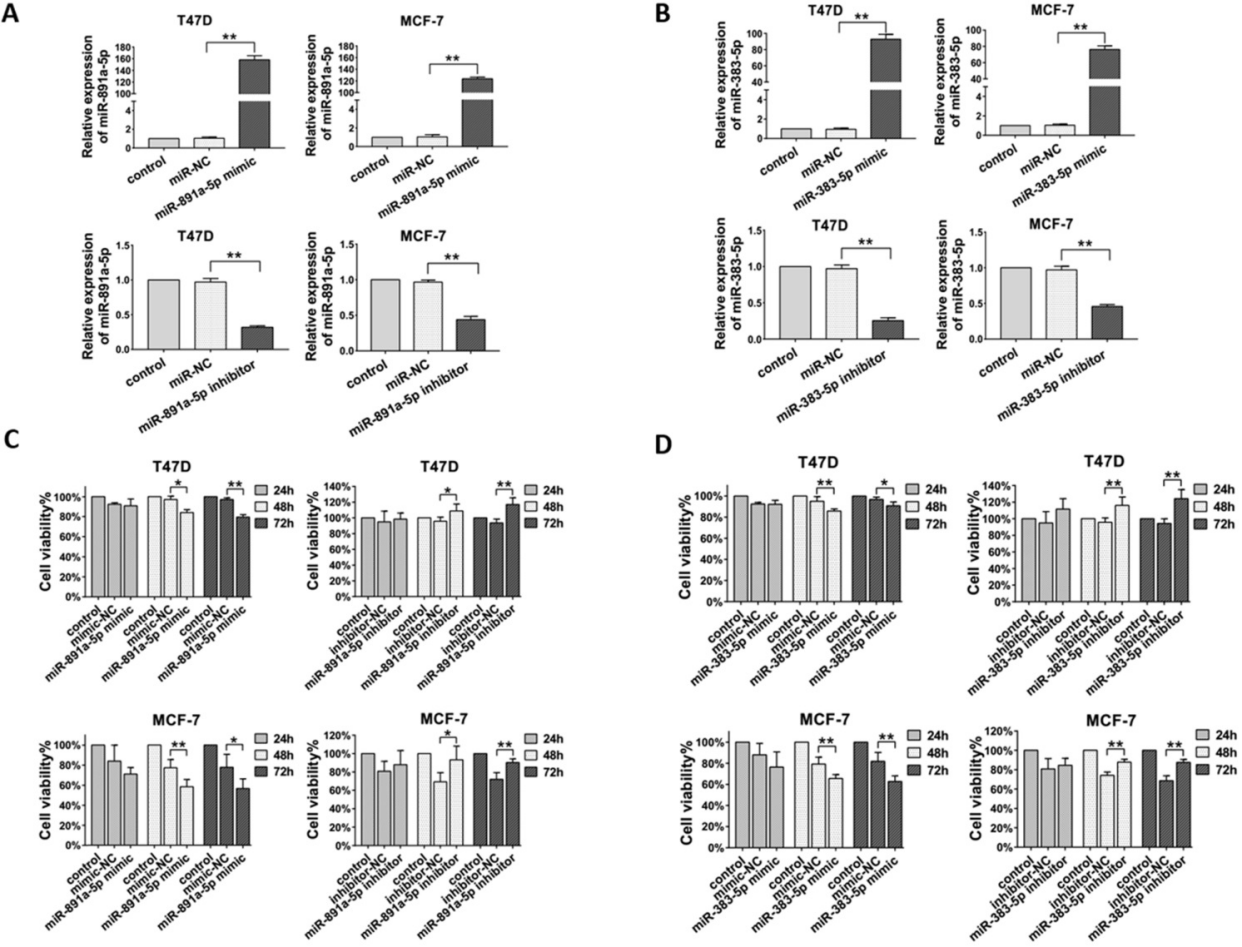
** Cytotoxicity of miR-891a-5p and miR-383-5p on breast cancer cells.** (A-B) After T47D and MCF7 cells were respectively transfected with miR-NC (negative control), miR-891a-5p mimic (A), miR-891a-5p inhibitor (A), miR-383-5p mimic (B) and miR-383-5p inhibitor (B) for 48 hrs, mRNA expression level of miR-891a-5p (A) ormiR-383-5p (B) was detected by real-time PCR. (C-D) After T47D and MCF7 cells were respectively transfected with miR-NC (negative control), miR-891a-5p mimic (C), miR-891a-5p inhibitor (C), miR-383-5p mimic (D) and miR-383-5p inhibitor (D) for 24, 48 or 72 hrs, cell viability was detected by MTT assay.(The data represent at least three independent experiments. *P<0.05, **P<0.01.

Next, in order to determine the effect of miR-891a-5p and miR-383-5p on breast cancer cells, their corresponding synthetic miRNA mimics and inhibitors were applied for this study. In addition, estrogen receptor-positive breast cancer T47D and MCF7 cells were used in this study. As shown in **Figure 3A**, transfection of miR-891a-5p mimic upregulated, whereas transfection of miR-891a-5p inhibitor downregulated the mRNA expression level of miR-891a-5p in both T47D and MCF-7 cells. Similar results were found for miR-383-5p (**Figure 3B**). Furthermore, miR-891a-5p and miR-383-5p mimics significantly inhibited whereas their inhibitors promote the proliferation of T47D and MCF-7 cells in a time-dependent manner, respectively (**Figures 3C and 3D**). These results showed that miR-891a-5p and miR-383-5p restrained the proliferation of breast cancer cells.

### miR-891a-5p but not miR-383-5p suppressed breast cancer cells migration

**Figure 4:**
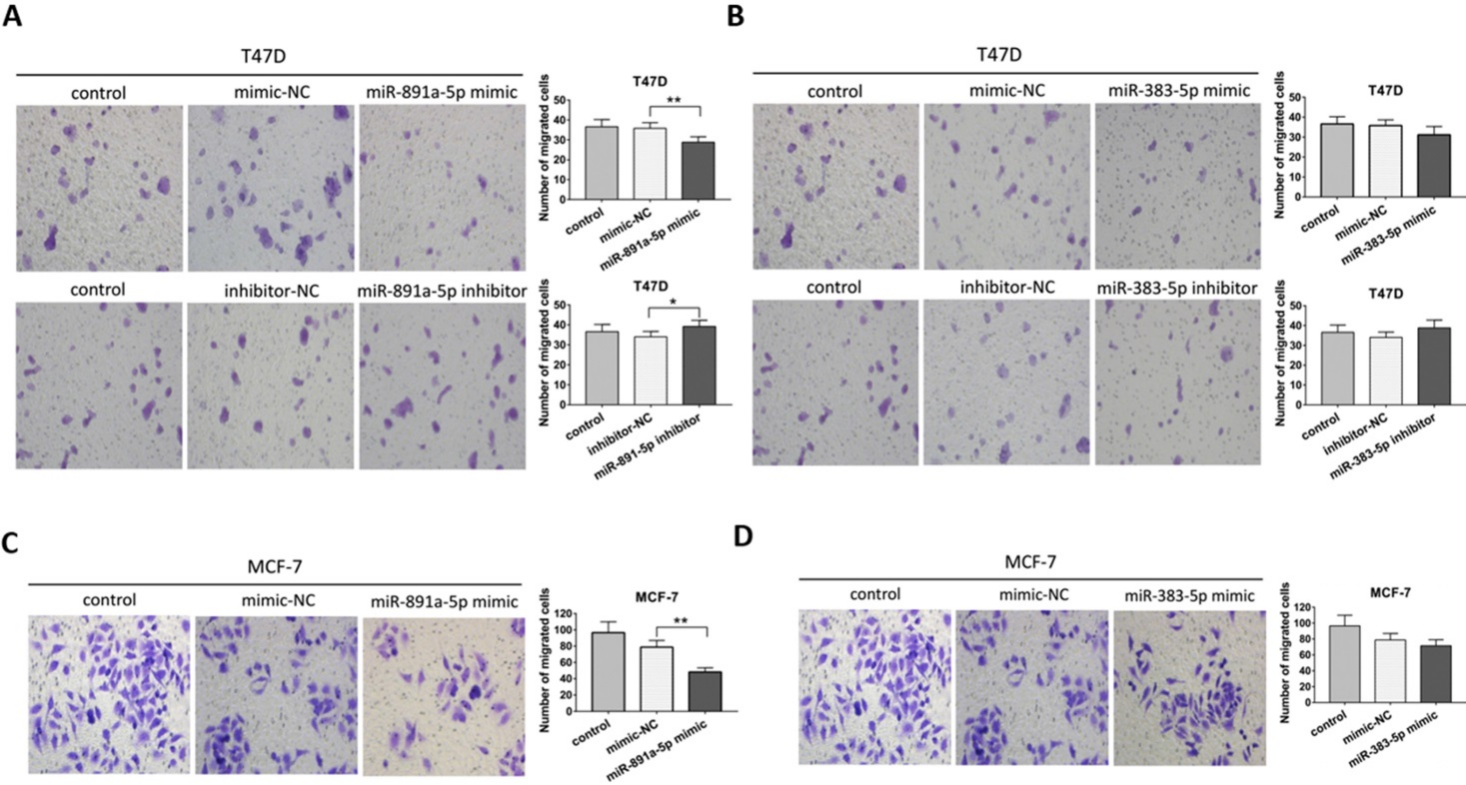
** miR-891a-5p but not miR-383-5p suppressed the migration of breast cancer cells.** (A-B)After T47D cells were respectively transfected with miR-NC (negative control), miR-891a-5p mimic (A), miR-891a-5p inhibitor (A), miR-383-5p mimic (B) and miR-383-5p inhibitor (B) for 48 hrs, cell migration was measured by Transwell migration experiment. (C-D) After MCF7 cells were respectively transfected with miR-NC (negative control), miR-891a-5p mimic (C), and miR-383-5p mimic (D) for 48 hrs, cell migration was measured by transwell migration experiment.The data represent at least three independent experiments. *P<0.05, **P<0.01.

Transwell migration assay was applied for further determination on the pro-metastatic ability of miR-891a-5p and miR-383-5p in breast cancer cells. As presented in **Figure 4A**, miR-891a-5p mimic significantly inhibited, whereas its inhibitor promoted the migration of T47D cells. However, neither miR-383-5p mimic nor inhibitor affected the migration of T47D cells (**Figures 4B**). Moreover, miR-891a-5p mimic but not miR-383-5p mimic halted the migration of MCF7 cells (**Figures 4C and 4D**). These data suggested that miR-891a-5p but not miR-383-5p suppressed breast cancer cells migration, implying miR-891a-5p is a valuable target for breast cancer therapy.

### ADAM10 is a downstream target of miR-891a-5p

**Figure 5:**
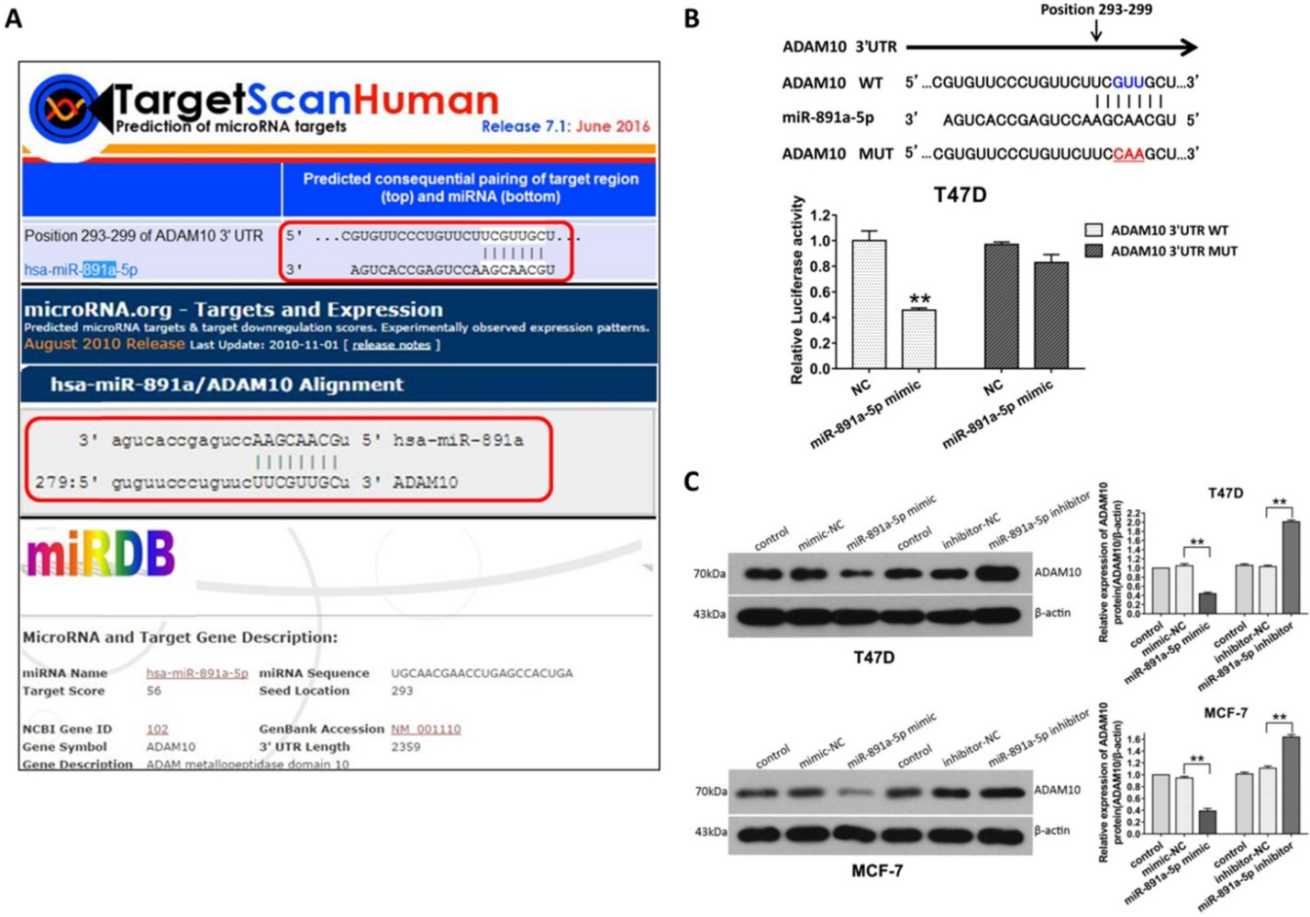
** ADAM10 is a downstream target of miR-891a-5p.** (A) The sequences of ADAM10 3' UTR bound by miR-891a-5p predicted through online websites Targetscan, microRNA.org, and miRDB. (B)T47D cells were co-transfected with miR-NC ormiR-891a-5p mimic and the firefly luciferase reporter plasmid harboring full-length 3'UTR of ADAM10 (WT) or Mutant (Mut) in which the target site of miR-891a-5p is mutated. Then the cell lysate was collected for luciferase activity detection. (C) After T47D and MCF7 cells were respectively transfected with miR-NC (negative control), miR-891a-5p mimic and miR-891a-5p inhibitor for 48 hrs, cell lysate was collected for western blot analysis using the antibodies as indicated. Left panel: immunoblot; Right panel: quantitation. The data represent at least three independent experiments. **P<0.01.

In order to further identify the gene targeted by miR-891a-5p, in silico analysis of miRNAs target gene through online computational algorithms (Targetscan, microRNA.org, miRDB, and RNA22-HAS) was applied. The result revealed that A Disintegrin and metalloproteinase domain-containing protein 10 (ADAM10) is the downstream target for miR-891a-5p (Data not shown).As depicted in **Figure 5A**, position 293-299 of ADAM103'UTR is the putative binding site formiR-891a-5p.To further confirm that miR-891a-5p regulates ADAM10 expression through this binding site, a firefly luciferase reporter plasmid harboring full-length 3'UTR of ADAM10 was constructed. Interestingly, miR-891a-5p mimic limited the luciferase activity in T47D cells which were transfected with ADAM10 3'UTR luciferase vector (**Figure 5B, WT**). In contrast, miR-891a-5p mimic did not significantly affect the luciferase activity in T47D cells when transfecting with ADAM10 3'UTR luciferase vector mutant in which the target site of miR-891a-5p is mutated (**Figure 5B, Mut**). Moreover, western blot analysis showed that upregulation of miR-891a-5p by mimic decreased whereas down- regulation of miR-891a-5p by inhibitor enhanced the expression level of ADAM10 in both T47D and MCF7 cells (**Figure 5C**). These results indicated that miR-891a-5p inhibited ADAM10 expression by directly binding to its 3'UTR.

### miR-891a-5p impeded breast cancer cells proliferation and migration through downregulation of ADAM10 expression

**Figure 6:**
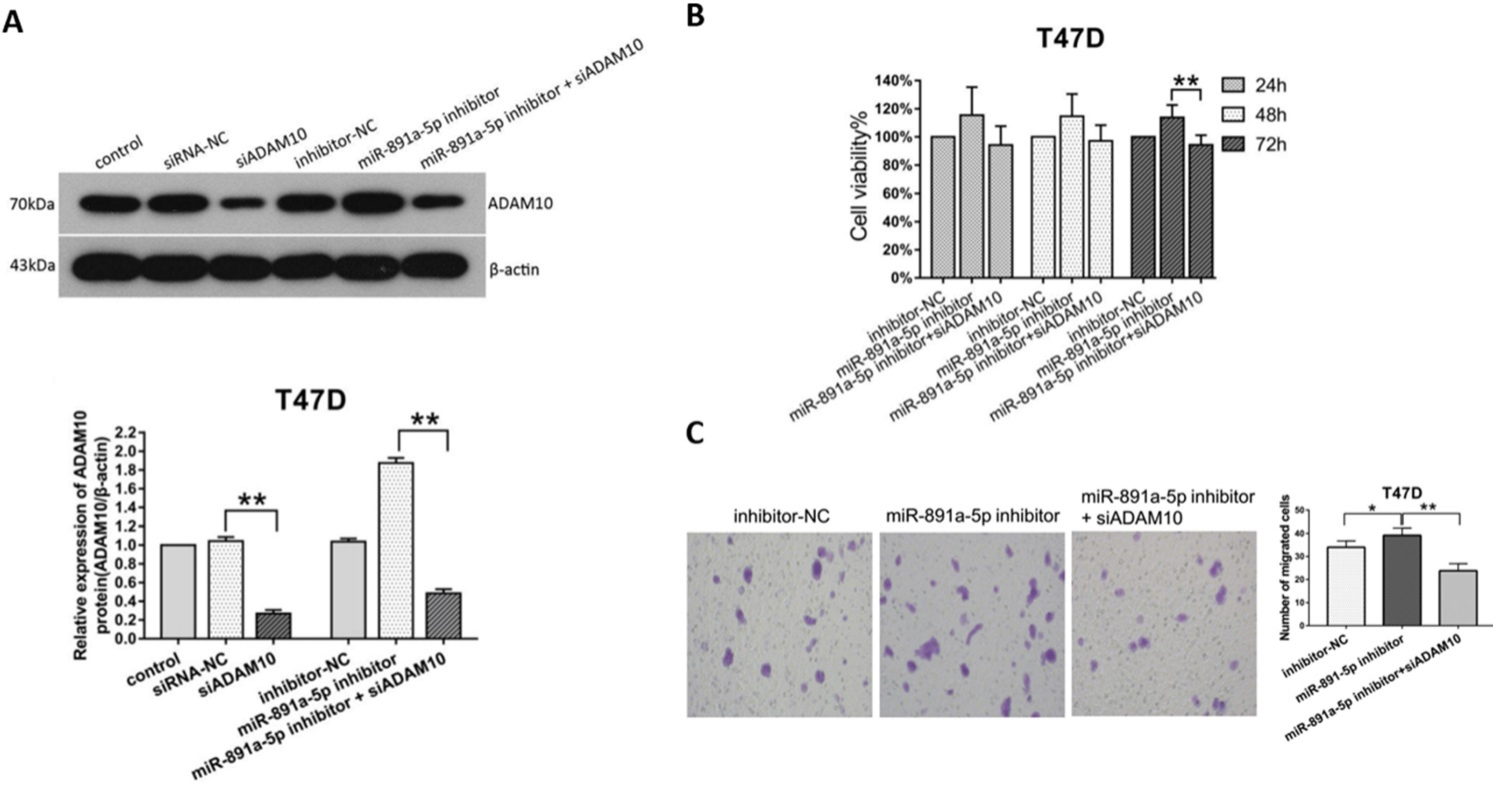
** Silencing of ADAM10 rescued the inhibitory effect of the low miR-891a-5p expression on breast cancer cells proliferation and migration.** (A) After T47D cells were respectively transfected with miRNA or siRNA as indicated for 48 hrs, cell lysate was obtained for western blot analysis using the antibodies as indicated. Upper panel: immunoblot; Lower panel: quantitation. (B-C) After T47D cells were respectively transfected with miRNA or siRNA as indicated for 24, 48 or 72 hrs, cell viability was detected by MTT assay (B), and cell migration was measured by Transwell migration experiment (C).The data represent at least three independent experiments. *P<0.05, **P<0.01.

To ascertain the cytotoxicity of miR-891a-5p on breast cancer cells through downregulation of ADAM10 expression, MTT, and Transwell migration assays were conducted. Western blot analysis disclosed that the increment of ADAM10 expression in T47D cells induced by miR-891a-5p inhibitor treatment was blocked by co-treatment with siRNA targeting ADAM10 (**Figure 6A**). Moreover, co-administration with miR-891a-5p inhibitor and siRNA targeting ADAM10 hindered the up-regulation of cell proliferation (**Figure 6B**) and migration (**Figure 6C**) of T47D cells elicited by miR-891a-5p inhibitor treatment alone. These results confirmed that miR-891a-5p impeded breast cancer cells proliferation and migration through downregulation of ADAM10 expression.

**Figure 7:**
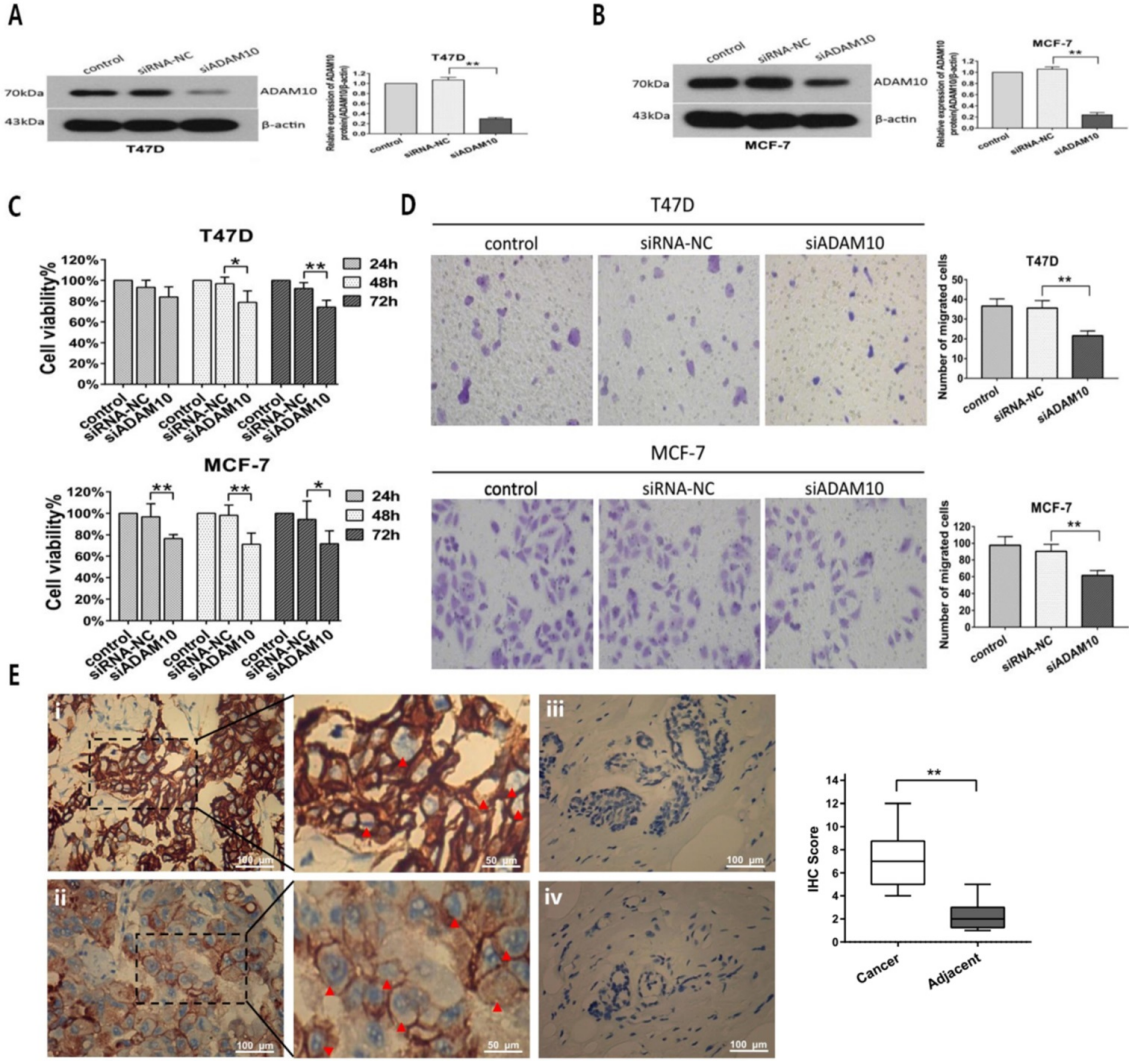
** Silencing ADAM10 inhibited breast cancers growth and migration.** (A-B) After T47D (A) and MCF7 (B) cells were respectively transfected with siRNA-NC (negative control) and siADAM10 for 48 hrs, cell lysate was collected for western blot analysis using the antibodies as indicated. Left panel: immunoblot; Right panel: quantitation. (C-D) After T47D and MCF7 cells were respectively transfected with siRNA-NC and siADAM10 for 24, 48 or 72 hrs, cell viability was detected by MTT assay (C), and cell migration was measured by Transwell migration experiment (D).The data represent at least three independent experiments. (E) The expression ADAM10 in breast tissues was detected using immunohistochemistry. (i) and (ii): Positive expression shows brown-yellow particles distributed in the membrane and cytoplasm of the cells (red arrowhead) in breast cancer tissues; (iii) and (iv): Negative expression in adjacent normal breast tissues (SP staining × 200). *P<0.05, **P<0.01.

To further investigate the effect of ADAM10 on breast cancer cells proliferation and migration, MTT and Transwell migration assays were applied after T47D and MCF7 cells were transfected with siRNA targeting ADAM10. Silencing ADAM10 by siRNA (**Figures 7A and 7B**) in T47D and MCF7 cells exhibited inhibitory activity on cell viability in a time-dependent manner (**Figure 7C**). Moreover, Knockdown of ADAM10 suppressed the migratory capacity of T47D and MCF7 cells (**Figure 7D**). Interestingly, high expression levels of ADAM10 were found in breast cancer tissues as compared to the adjacent normal breast tissues (Figure 7E). These results elucidated that silencing ADAM10 inhibited breast cancers growth and migration, suggesting that ADAM10 is a potential target for HR-positive breast cancer therapy.

## Discussion

In this study, upon analysis of GSE59829 dataset and our clinical cohort, we identified miR-891a-5p and miR-383-5p were the potential prognostic markers for metastatic human HR-positive breast cancers. Mechanistic investigation unraveled miR-891a-5p but not miR-383-5p impeded the expression of the downstream target ADAM10 by directly binding to its 3'UTR, leading to the inhibition of breast cancer cells proliferation and migration. The results advocate that miR-891a-5p is the promising target for the prognosis and therapeutics of HR-positive breast cancers.

Our clinical data furnish evidence that the low expression levels of miR-891a-5p and miR-383-5p in metastatic human HR-positive breast tumors are associated with low distant metastasis-free survival (DMFS) in patients. Upon the advanced high-throughput microarray analysis technology, high expression levels of miRNAs associated with the recurrence and metastases of HR-positive breast cancers have been characterized in previous studies. Zhou et al. revealed that high expression level of miR-9 in tumor tissues can be used to predict the risk of local recurrence and overall survival (OS) of HR-positive breast cancer 16. In addition, miR-190b was found highly expressed in HR-positive breast cancer tissues and correlated with progression-free survival (PFS) and OS in patients with HR-positive breast cancer 17. Eissa et al. used clinical histology validation, and multivariate survival analysis confirmed that high expression of miR-221 in breast cancer tissue was correlated with the poor prognosis in patients with HR-positive breast cancer 18. In current report, we identify that low expression levels of miR-891a-5p and miR-383-5p were in metastatic human HR-positive breast tumor tissues (**Figure 1**) and contribute to poor DMFS in patients (**Figure 2**) in our clinical data. The results were inconsistent with the findings obtained from the analysis of GSE59829 dataset (**Figures S2 and S3**). This discrepancy might be due to several reasons. First, different postoperative treatments were carried out. The breast cancer patients enrolled in GSE68373 dataset did not receive any adjuvant treatment. In contrast, the patients enrolled in our study underwent adjuvant therapy (endocrine therapy + chemotherapy). Second, microarray detection still has some limitations. In GSE68373 dataset, the researchers detected the miRNAs expression by conventional high-throughput microarray detection method. It is theoretically difficult to distinguish between miRNAs with tiny differences and precursor miRNAs and mature miRNAs with the same sequence. It is necessary to confirm to the microarray results using *in vitro* bench experiments 19. Third, the patients enrolled in GSE68373 were mainly European Caucasian (Italy), while the patients enrolled in our clinical study were Asian.

In accordance with clinical results, our observations that miR-891a-5p inhibits breast cancer T47D and MCF-7 cells proliferation and metastasis imply that miR-891a-5p is a potential therapeutic target for HR-positive breast cancers. It is reported that miR-383-5p suppressed the proliferation and migration of various tumors including cervical cancer 20, pancreatic cancer 21, and breast cancer cells 22. In contrast, miR-891a-5p is a newly characterized miRNA located on the Xq27 chromosome 23 and its role in cancers is barely reported. In this study, our investigations demonstrate that miR-891a-5p can inhibit the proliferation and migration of breast cancer T47D and MCF-7 cells (**Figures 3 and 4**), hinting that miR-891a-5p works as the suppressor of HR-positive breast cancer onset and metastasis. Recently, multiple miRNAs were found involved in the invasion and metastasis of breast cancers. MiR-10b, which as highly expressed in human breast cancer cell line MDA-MB-231, promoted the invasion and migration of breast cancer cells^24^. MiR-125b has been found to constrain breast cancer cell proliferation, invasion and migration by targeting erythropoietin (EPO) and EPO receptors and ERBB2 25-27. Furthermore, miR-17/20a inhibited breast cancer metastasis through activating NK cells by targeting Mekk2^28^.Our findings in this study provide novel promising agents for breast cancer intervention using miR-891a-5p.

Our discovery indicates that ADAM10 is a downstream target for miR-891a-5p. MiRNAs are wide-distributed, single-stranded and non-coding RNA molecules. They were involved in the regulation of gene expression by binding to the 3'-untranslational region (UTR) of target genes and dampening the translation of mRNAs (Bartel, 2004). Similar to other miRNAs targeting downstream gene expression deciphered by other researchers 28, 29, our studies disclose that ADAM10 is a downstream target for miR-891a-5p, which is for the first time characterized by us using bioinformatics method, dual-luciferase reporter assay, and western blot analysis (**Figure 5**). Our studies also reveal that miR-891a-5p impeded breast cancer cells proliferation and migration through downregulation of ADAM10 expression (**Figure 6**), further signifying that miR-891a-5p is a valuable target for breast cancer therapy. Besides the downregulation of ADAM10 by miR-655-3p in inhibitory activity on the proliferation, invasion, and metastasis of hepatoma cells 30, our investigations suggest a new insight into the regulation of ADAM10 expression by miR-891a-5p in breast cancer cells.

ADAM10 is a member of the type I transmembrane ADAM protein family which consists of disintegrin and metalloproteinase domains. The metalloproteinase domain hydrolyzes various growth factors and adhesion molecules, and degrades the extracellular matrix, while the disintegrin domain is a ligand for integrin proteins and participates in cell adhesion signaling pathways 31, 32. Previous studies reported that ADAM10 was up-regulated in head and neck squamous cell carcinoma 33 and liver cancer 34, hypopharyngeal carcinoma 35 and tongue cancer^36^, and promoted the proliferation, invasion, and migration of tumor cells. Although ADAM10 was up-regulated in the triple-negative breast cancer cell line MDA-MB-231 37, the role of ADAM10 in the regulation of HR-positive breast cancer cells proliferation and migration is still unclear. In this report, we present evidences that ADAM10 is highly expressed in HR-positive breast cancer tissue and silencing ADAM10 can significantly inhibit the proliferation and migration of HR-positive breast cancer cells (**Figure 7**). This result is in accordance with previous findings in other malignant tumors and ascertains that ADAM10 is a potential candidate for breast cancer therapeutics.

## Conclusions

Our clinical investigation evinces that low expression levels of miR-891a-5p and miR-383-5p were found in metastatic human HR-positive breast tumors, which associated with poor prognosis evidenced by low DMFS in patients. *In vitro* experiments validate that miR-891a-5p hindered the expression of their shared downstream target ADAM10 by directly binding to its 3'UTR, resulting in the inhibition of breast cancer cells proliferation and migration. These observations prove the possible utility of miR-891a-5p as promising prognostic biomarkers and therapeutic targets for HR-positive breast cancer.

## Supplementary Material

Supplementary figures.

## Figures and Tables

**Table 1:** The detailed information and characteristics of breast tumors collected from patients the First Affiliated Hospital of China Medical University

Variables	Metastasis (n=28)	Without metastasis (n=34)	*p*
**Age(years) , n(%)**			0.816
≤ 50	19 (67.86)	24 (70.59)	
>50	9 (32.14)	10 (29.41)	
**T stage, n(%)**			0.779
T1	13 (46.4)	17 (50.0)	
T2	15 (53.6)	17 (50.0)	
**Adjuvant therapy, n (%)**			0.705
Chemotherapy	19 (67.9)	20 (58.8)	
Hormone therapy	27 (96.4)	34 (100.0)	
**Molecular subtyping,n(%)**			0.424
Luminal A	8 (28.6)	13 (38.24)	
Luminal B	20 (71.4)	21 (61.76)	
**Histotype ,n(%)**			0.737
IDC	23 (82.1)	29 (85.29)	
rests (ILC, DCIS-MI, mucinous carcinoma)	5 (17.9)	5 (14.71)	
**Metastatic sites, n(%)**			NA
Lung metastasis	9	NA	
osseous metastasis	12	NA	
hepatic metastases	7	NA	
**Follow-up time (months)**			<0.001
Min-Max	6-86	59-131	
Median	46	81	

Note: Luminal A: ER and PR positive (≥1%) , HER-2 negative, Ki67 low expression (<14%,) ; Luminal B: ER or PR positive (≥1%), HER-2 negative, Ki67 high expression (≥14%). NA, Not Applicable.
