# Supplementary material for: MiR-891a-5p as a prognostic marker and therapeutic target for hormone receptor-positive breast cancer
Source: J Cancer. 2020 Apr 6;11(13):3771–82. doi: 10.7150/jca.40750 (PMC7171503; doi:10.7150/jca.40750)

Figure S1 Thermograms representing differential expression of microRNAs in breast cancers from patients in GSE59829 dataset

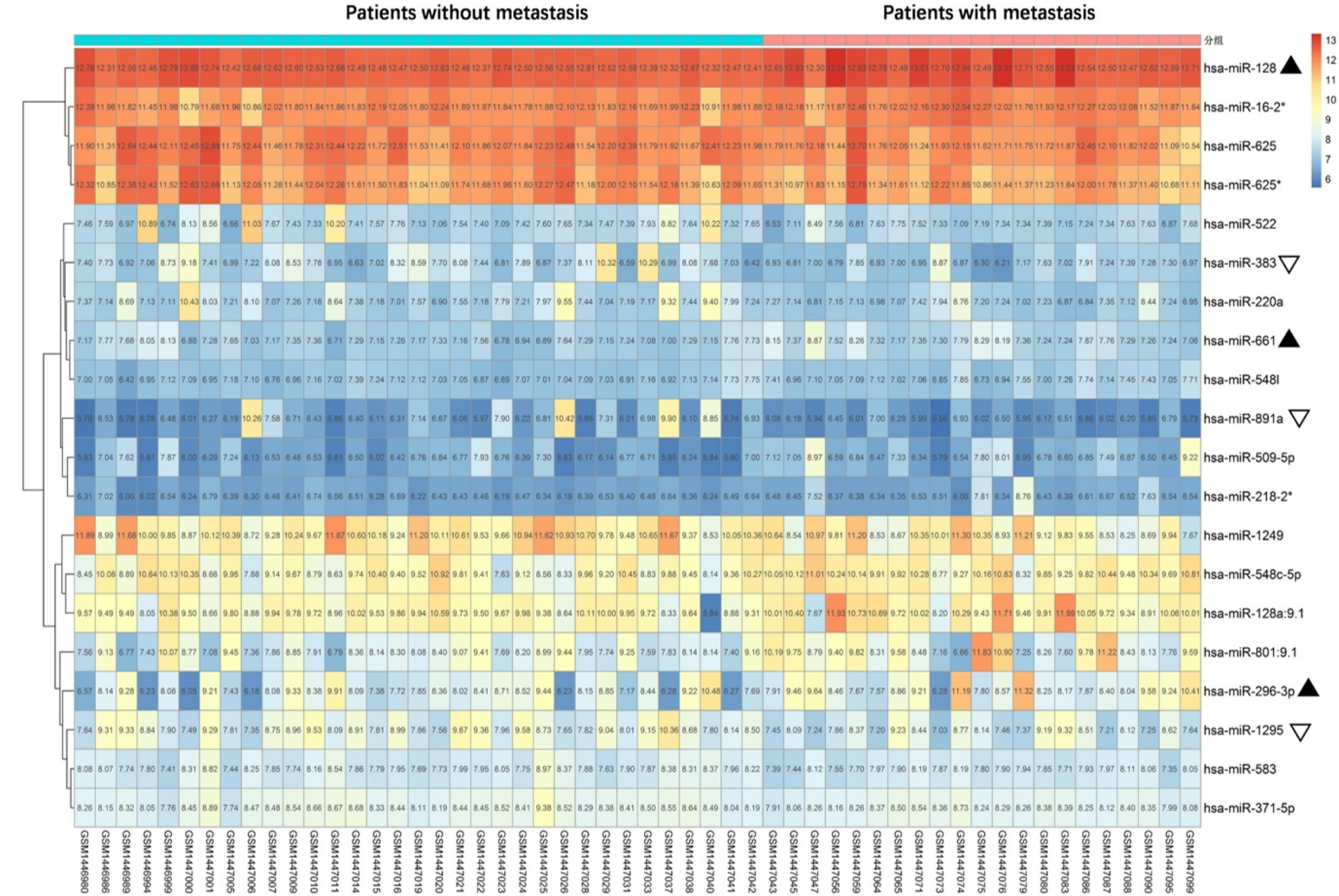

Figure S2 The expression level of microRNAs in breast cancers from patients with or without metastasis in GSE59829 dataset

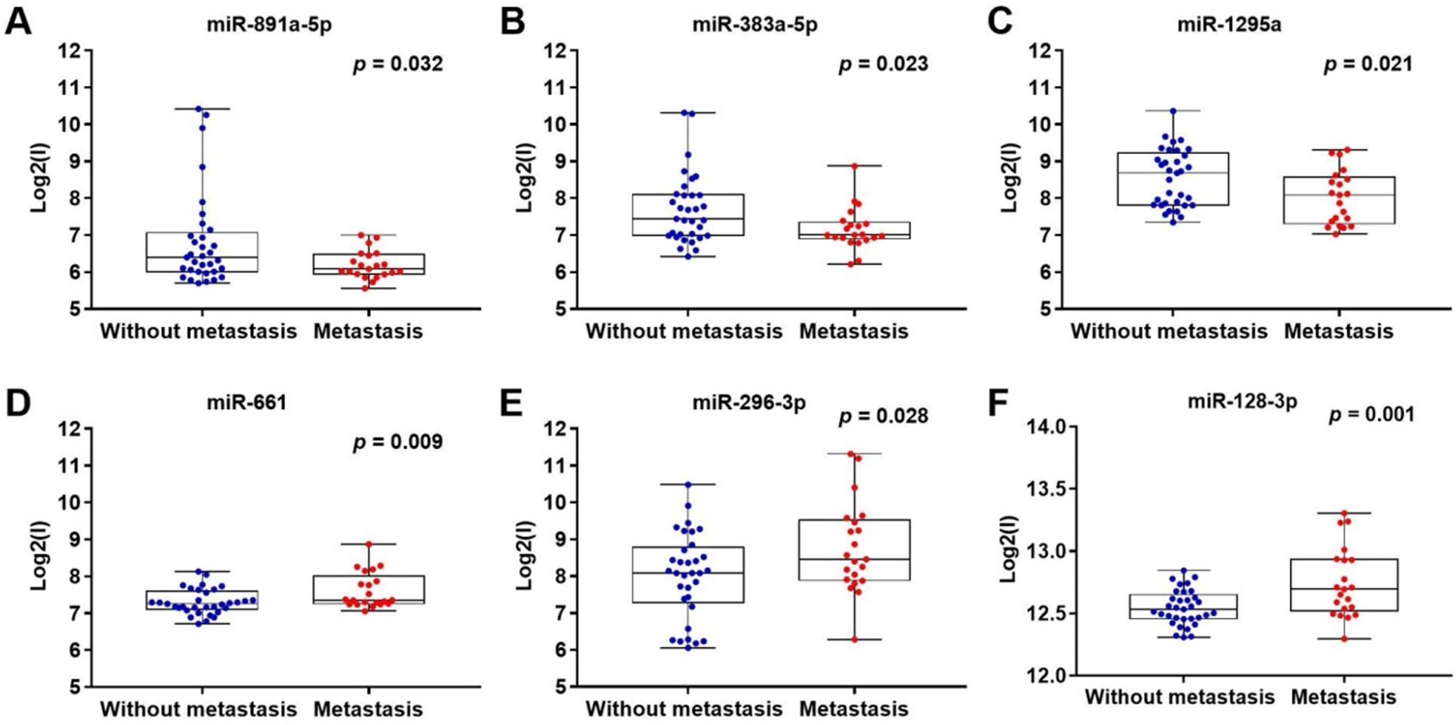

Figure S3 The correlation between the expression level of microRNAs and distant metastasis free survival of patients with breast cancer in GSE59829 dataset

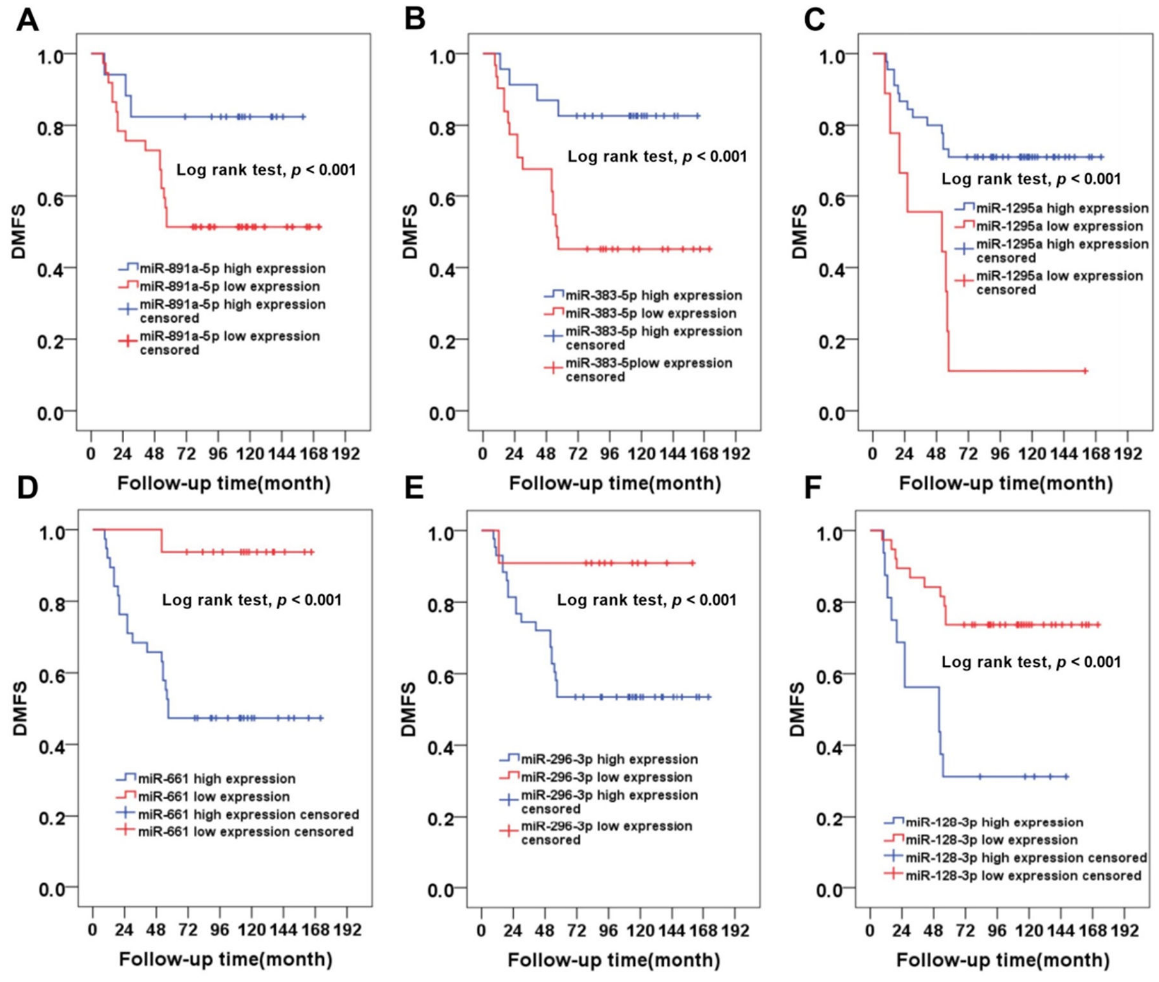

Supplement: Supplementary file 1 — Supplementary figures. [file jcav11p3771s1.pdf]
